# Factors related to the implementation and scale-up of physical activity interventions in Ireland: a qualitative study with policy makers, funders, researchers and practitioners

**DOI:** 10.1186/s12966-023-01413-5

**Published:** 2023-02-14

**Authors:** Joey Murphy, Fiona Mansergh, Grainne O’Donoghue, Femke van Nassau, Jemima Cooper, Caera Grady, Niamh Murphy, Enrique Garcia Bengoechea, Marie H. Murphy, Benny Cullen, Catherine B. Woods, the I-PARC

**Affiliations:** 1 Centre for Exercise, Nutrition & Health Sciences, School for Policy Studies, University of Bristol, Bristol, UK; 2 Department of Health, Healthy Ireland, Block 1, Miesian Plaza, 50-58 Lower Baggot Street, Dublin, Ireland; 3 School of Public Health, Physiotherapy & Sports Science, University College Dublin, Dublin, Ireland; 4 Amsterdam UMC, Vrije Universiteit Amsterdam, Department of Public and Occupational Health, Amsterdam Public Health Research Institute, Amsterdam, The Netherlands; 5 Physical Activity for Health Research Cluster, Department of Physical Education and Sport Sciences, University of Limerick, Limerick, Ireland; 6 Department for Health, University of Bath, Bath, UK; 7 Department of Sport and Exercise Science, Waterford Institute of Technology, Waterford, Ireland; 8 Research & Innovation Unit, Sport Ireland, Dublin, Ireland; 9 Centre for Exercise Medicine, Physical Activity and Health, Sports and Exercise Sciences Research Institute, Ulster University, Jordanstown Campus, Coleraine, UK

**Keywords:** Consolidated framework for implementation research, Thematic analysis, Facilitator, Barrier, Perspectives

## Abstract

**Background:**

Current literature reports a gap between development of effective interventions to promote physical activity and the systematic uptake into real-world settings. Factors relating to implementation and scale-up of physical activity interventions have been examined, however the perspectives of multiple stakeholders from different domains are not well researched. The purpose of this study was to examine the perceived factors related to physical activity intervention implementation and scale-up in different domains from different stakeholders on the island of Ireland.

**Methods:**

Practitioners, researchers, funders and policy makers in Ireland were invited to take part in a semi-structured interview exploring factors related to the implementation and scale-up of eleven different physical activity interventions. A thematic analysis was conducted to identify factors related to the implementation and scale-up of the included interventions. The data collection and analysis were guided by the Consolidated Framework for Implementation Research.

**Results:**

Thirty-eight participants took part in the interviews which identified factors related to 1) intervention planning and practical considerations; 2) organisational structures, staffing and resources related to delivery; 3) reflection, evaluation and updating of the intervention; and 4) practical consideration related to scale-up. Furthermore, participants referred to the ongoing commitment, engagement, and support needed throughout the implementation process.

**Conclusions:**

Future research and practice needs to consider how different factors are experienced at different implementation stages and by the different stakeholder groups involved. The findings highlight multiple inter-related factors that influence the implementation and scale-up of physical activity interventions, but also identifies many strategies that can be utilised to aid future successes.

**Supplementary Information:**

The online version contains supplementary material available at 10.1186/s12966-023-01413-5.

## Background

Participation in regular physical activity (PA) has been shown to reduce the global burden of non-communicable diseases [1] and strengthen the immune system, suggesting a benefit in response to viral communicable diseases [2]. It is also important to acknowledge that regular PA is associated with quality of life [3], mental health [4–6], and economic benefits [7]. The recent World Health Organization (WHO) Guidelines on PA and Sedentary Behaviour provide recommendations for children, adolescents, adults, older adults as well as for subpopulations, such as pregnant and postpartum women, and people living with chronic conditions or a disability [8]. Despite these updated recommendations, the associated benefits, and ever-growing number of interventions developed and implemented to promote PA [9], global estimates show that one in four (28%) adults [10] and more than three-quarters (81%) of adolescents [11] do not meet the recommendations for aerobic PA. This is reflected in Ireland where data show 87% of children and adolescents [12], 41 – 46% of adults [13, 14] and 67% of older adults [15] do not meet recommended PA guidelines, despite the publication of a national PA plan [16].

Current literature reports a gap between development of effective interventions to promote PA and the systematic uptake of these interventions in real-world settings [17, 18]. To bridge this gap, Durlak and Dupre (2008) suggest that there is a critical need to understand factors related (i.e. determinants) to programme implementation [17], an emerging area of research that aims to promote this systematic uptake of interventions in real-world settings [19]. Implementation can be defined as the process of integrating an intervention into practice within a specific setting [20, 21], with frameworks available to guide our understanding of factors related to implementation and scale-up [22]. One such framework, the Consolidated Framework for Implementation Research (CFIR), combines key constructs from many published implementation models and theories into a single framework [21] helping understand determinants of implementation. The CFIR framework presents 39 constructs across 5 domains; 1) intervention characteristics, 2) characteristics of the individuals; 3) inner setting, 4) outer setting, and 5) processes of implementation. Such frameworks have been used to guide our understanding of implementing PA interventions and increase our understanding of the facilitators and barriers experienced during ‘real world’ implementation, ultimately contributing to developing and tailoring strategies that aid successful implementation [22, 23].

While several reviews have examined factors relating to implementation of PA interventions [23–27], the perspectives of multiple stakeholders (i.e., policy makers, funders, researchers, and practitioners) from different domains are not well understood. Studies have explored the perspectives of Australian policy-makers, practitioners and researchers experience regarding the scale-up of population health interventions [18, 28], finding that these stakeholders had different, but complementary roles in the process of scaling up an intervention [18]. Building on previous work, further research gaining insight from different stakeholders involved across the whole implementation process can provide a more complete picture regarding the important factors that influence implementation and scale-up. Understanding what enables “successful” implementation within different domains will also build on work in Ireland that identifies good practice examples for promoting PA using a systems approach [29]. To gain this understanding, qualitative methods are recognised as important approaches for developing the evidence base in PA and research due to the in-depth insight of key stakeholder’s perceptions they generate [30]. Thus, the purpose of this study was to qualitatively examine the perceived factors related to PA intervention implementation and scale-up in different domains from the perspectives of policy makers, funders, researchers, and practitioners in Ireland guided by the CFIR framework.

## Methods

### Selecting example interventions

This research was conducted through the Irish Physical Activity Research Collaboration (I-PARC), which is comprised of national Government Departments (*n* = 4), state agencies (*n* = 5) and research institutions (*n* = 6) [31] on the island of Ireland. Interventions were defined by the project team as “any form of PA service, programme or strategy aimed at increasing PA levels with general or special populations”. Interventions were selected if they were seen as an example of good practice by the project team, which meant they had proven efficacy for increasing PA in a controlled research setting (evidence-based practice), originated outside the scientific realm but proven to work in practice (practice-based evidence) [32], or a combination of both. These “example interventions” were selected through a 3-stage process:

1. A short form was administered to those working in various sectors to gain an understanding of the interventions currently being delivered for the promotion of PA across the island of Ireland.
2. Responses were reviewed by all members of the I-PARC team to ensure interventions represented a range of settings and target groups. The settings included transport, urban design, healthcare, education/work, community-wide, communication/mass-media, and sport and recreation. The target groups included general population, people with disabilities, clinical populations and people with low socio-economic status across the lifespan.
3. Once responses were reviewed, two consensus meetings were held to select the final interventions which would best represent good practice across the settings and target groups.

In total, 49 interventions were identified through the form with 11 selected as examples of good practice for inclusion in this qualitative study.

### Selection of stakeholders within the example interventions

Purposeful sampling techniques were used as expert knowledge and insight was sought from policy makers (PM), funders (FU), service providers (SP), service coordinators (SC) and researchers (RE) across the selected interventions. Definitions of these stakeholder groups are provided in Table 1. In total, the project team aimed to involve both a service provider and coordinator from each intervention selected (*n* = 22) and five policy makers, funders and researchers involved across the interventions (*n* = 15). Like the interventions, the stakeholders were identified by members of the I-PARC project team with efforts made to ensure all those selected covered the implementation of interventions in the settings and populations mentioned earlier.

**Table 1:** Definition used for each stakeholder group

Stakeholder Group	Definition Used
Policy Maker (PM)*	Individuals involved in agenda setting, fund surveillance/monitoring, and high-level support and advocacy for physical activity participation at a political level
Funder (FU)	Those that are involved in the provision of funding to develop run and scale-up interventions for increasing physical activity
Service Coordinator (SC)	Those that oversee the running of the intervention. They may not directly engage with the participants but have knowledge of the development of such intervention and all the components included
Service Provider (SP)	Those involved with the delivery of an intervention and engage face-to-face with participants. This can include the following personnel: Sports Development Officer, Club/Group Coach, External Contractor, Volunteer, Health and Fitness Instructor, Clinician, Primary Care Personnel
Researcher (RE)	Individuals involved in research elements of the intervention, including pilot testing, feasibility trails, efficacy and/or effectiveness testing and evaluations

Abbreviations used for each stakeholder group can be seen in the above table. *In some cases, depending on funding source, policy makers can also be seen as a funder

### Survey

Prior to the interviews, service coordinators were sent an online survey (Qualtrics, Provo, UT). The purpose of this survey was to gain insight into each intervention and generate prompts that would help to guide the interviews. The survey used open and closed questions based on the five domains of the CFIR [21]. This was piloted with two service coordinators to assess applicability for the target population and feedback was used to revise questions and responses. The final survey questions were approved by all project team members and are available in Supplementary File 1. Once responses were received, they were reviewed before conducting the interview.

### Interviews

A semi-structured interview was conducted with each participant (*n* = 38). The interviews comprised of open-ended questions including prompts and closed questions to ensure relevant data were collected. The CFIR was chosen to help structure, extract and synthesise the findings as it is a commonly used framework within implementation research [22, 23, 27]. Where possible, interviews were conducted in person (*n* = 8). However, due to COVID-19 restrictions, the majority were conducted remotely by telephone (*n* = 30). Interviews lasted approximately 60-min each, were recorded using a Dictaphone (Olympus VN-541PC) and transcribed to ensure anonymity during analyses.

### Interview questions

Two sets of interview questions were created depending on the participant type to ensure questions were applicable for the stakeholders involved. Interview questions for the service coordinators and providers (Supplementary File 2) focused on intervention development and delivery, implementation strategies, and the role of different actors. Interview questions for the policy makers, funders and researchers (Supplementary File 3) were adapted from Milat and colleagues (2014) focusing on the decision processes, different actors and scaling up of the intervention [18]. Both interview scripts were piloted with a relevant stakeholder (i.e., one PM and one SC) and feedback provided was used to revise the questions asked. Interviews were structured in a repeatable way that would provide consistent data with reduced bias, increasing the reliability of the study [33]. Prompts, identified from the survey, were used throughout to help develop the content of each interview and further explore each intervention. A definition of terms such as implementation, scale-up, and decision processes were included in the interview guides and provided to participants during the interviews.

### Data analysis

Qualitative data collected through the interviews were analysed using a combination of inductive and deductive thematic approaches. This approach was chosen as it allowed for the interviews to be coded inductively first, before organising them deductively under the broad domains of the CFIR. Five members of the project team were involved in the data analysis, which was guided by the six phases for thematic analyses [34]. First, each member familiarised themselves with the data by reading a selected transcript from each stakeholder group. Each member open coded their interview transcript using an inductive approach, whereby no framework was used at this stage. Members then met virtually to organise common codes across interviews and theme them deductively under the CFIR domains. A “deviant case” category was used to manage codes that did not fit under the CFIR domains. The list of codes under each CFIR domain from this meeting were reviewed and any deviant cases were discussed regarding their placement within the framework or removal from the analysis (Supplementary File 4). This activity resulted in the generation of a “code book” (Supplementary File 5) which was used by the lead author to code all interviews through NVIVO (version 12). Once all interviews were coded, themes were identified under each of the CFIR domains. Members of the project team met three times to review and agree upon the themes identified. Involvement of different researchers throughout this process helped attenuate individual biases from the analysis and add credibility to these findings.

## Results

### Participants included

Thirty-eight participants (60.5% female) took part in the interviews. The proportion of stakeholder type was different than planned but deemed acceptable to meet the aims of the study. A description of the included interventions and associated stakeholders can be found in Table 2. The average length of interviews was 57 min (range: 38 – 76).

**Table 2:** Overview of stakeholders involved from the example interventions

N	Setting	Target Group	SP	SC	RE	FU	PM	Total
1	Sport and Recreation	General Population	2	1	1		1	**5**
2	Community	Adult Males	2	1	1	1		**5**
3	Community	Low SES	1	1		1	2	**5**
4	Education	Children	1	1	1		1	**4**
5	Education	Adolescents	1	1	1	1		**4**
6	Health	Clinical Population	1	1	1		1	**4**
7	Sport and Recreation	People with Disability	1	1		1		**3**
8	Community	General Population	1	1				**2**
9	Transport	General Population		1	1			**2**
10	Communication	Females		1			1	**2**
11	Health	Clinical Population	1	1				**2**
**Total**			11	11	6	4	6	**38**

*SP* Service provider, *SC* Service coordinator, *RE* Researcher, *FU* Funder, *PM* Policy maker

### Key findings

**Fig. 1:**
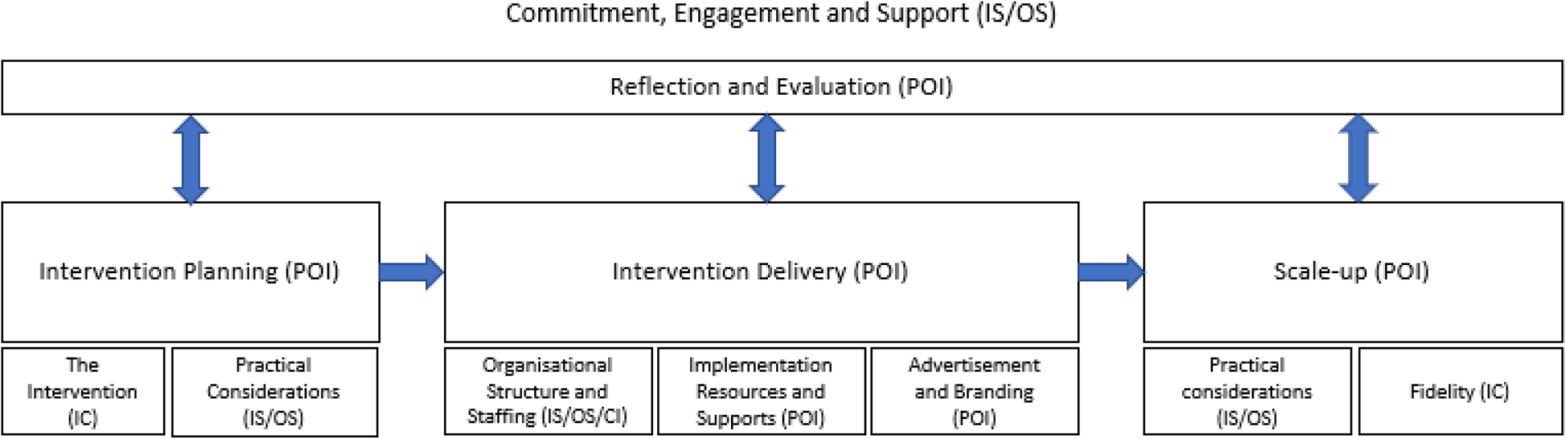
Model representing the structure of factors related to implementation of interventions and how they align to areas of the Consolidated Framework for Implementation Research. *IC *Intervention characteristics, *CI* Characteristics of the individuals, *IS* Inner setting, *OS* Outer setting, *POI* Processes of implementation

Throughout the interviews, participants spoke about different stages of implementation that include 1) planning, 2) delivery, 3) reflection and evaluation, and 4) scale-up. Reflection and scale up was seen to be important across all other stages of implementation. Furthermore, participants referred to the ongoing commitment, engagement, and support needed throughout the different stages of implementation. This was explored through themes relating to stakeholder engagement, funding support and recognition of achievements. Figure 1 shows the structure of themes identified as factors related to the implementation and scale-up of PA interventions, and how they align to the different domains of the CFIR [21].

### Stages of implementation

#### Intervention Planning

Participants stated *“you need an action plan” (SC 6)* when considering intervention implementation*.* Emphasis was placed on involving all relevant stakeholders from the beginning and planning how the intervention would be sustainable and scalable. Analysis revealed that planning components related to the intervention itself and other practical considerations positively influenced implementation.

### The intervention

When planning, participants felt it important to demonstrate intervention need. This need could be demonstrated from the top down (e.g., national data or policy gaps) or bottom up (e.g., appetite of the sector for an intervention). The importance of a “usable” intervention that was fit for the target population and compatible with current practice was also emphasised.

“You could have a great idea and it could be fantastic, but it just doesn’t fit in with the structure or the way your services are delivered. So, it has to be practical” (PM 2)

Cost of the intervention also needs consideration, with any funds generated through the intervention used to add further value to participants; “*Any money that it [intervention] makes is to go back into improving the service”* (SP 2).

Finally, participants noted the need for an overarching strategy that provides clear content and participant criteria that could be usable in different contexts.

“The core messages are very set, and you can progress them, you can give adaptations, you can jazz it up to make the class interesting” (SP 4)

### Practical considerations

Participants recommended several aspects across both inner and outer settings that need to be considered when planning. First, was the recommendation to assess the current context or system in which the intervention operates. Examples include the assessment of national policies, curriculum, or agenda (e.g., combat climate change) to identify what might impede or facilitate implementation.

“I might have a great idea, but if it’s not in national policy or it’s not a national agenda, then it’s going to be very difficult to make a case for it.” (PM 1)

It was noted that being aware of current regulations, such as health and safety legislation and guidelines, data protection regulations, and child protection measures can help ensure adequate systems and supports are available to deal with potential challenges. One service coordinator (SC11) mentioned the provision of an online *“Volunteer Hub”* as a source of useful documents for helping ensure Health and Safety and Child Protection were in place.

Within the local context, participants highlighted the need to observe that safe and accessible facilities and storage for equipment were available and to identify local stakeholders that could be potential partners to facilitate this. It was felt that failing to do this could lead to challenges such as lack of access to facilities due to competition with other local organisations, structure of facility (e.g. no disability access), or location of facility (e.g. lack of parking). Furthermore, interviewees noted that having knowledge of the location and target population could help overcome uncontrollable factors, such as the weather, competing priorities and public guidance (e.g., due to COVID-19).

“We started in September…It started getting darker…the Champions League was on…then the next week they [service users] wouldn’t come”. (SP3)

Finally, participants believed being aware of personnel required and the administration burden associated with intervention delivery at a local level (e.g. handling queries, completing evaluation) helped put systems in place to overcome challenges that lead to personnel dropout. Different stakeholders, especially service providers and coordinators, noted the importance of having personnel available to deal with administration and intervention delivery.

#### Intervention delivery

During the intervention delivery stage, an organisational structure with identification of personnel, use of advertisement strategies and availability of implementation resources were all seen as important for implementation.

### Organisational structure and staffing

An organisational structure within the lead organisation helped with implementation of the interventions. PM1 highlighted the importance of multi-disciplinary individuals in any steering group stating, “*different people representing their organizations bring their own expertise into that [steering group]”.* The steering group was seen as important during the initial implementation stages, but an organisational approach with layers (i.e., national, sub-national, and local) becomes more important when scaling up the intervention.

Within organisational structure, participants highlighted a need for those at all levels to be motivated, committed, and have a shared belief in the intervention. It was felt that motivation came from personal experience, consistent income or feeling a responsibility to create change, while belief in the intervention came from evidence it worked or benefited participants.

“All I have to do is go to a group and see the smiles on their faces. And once you've seen that, that's what kicked me off, is that I could see the impact that it had”. (SC 2).

Furthermore, several participants mentioned the need to identify a “champion” at political through to local level to support implementation. Coordinator 6 felt that a “champion” needs to be a *“big character that's quite engaged and that's very good at communicating with their members in the community that can bring other people on and delegate roles when needed”.*

Lack of support for administration requirements (i.e., paid by the hour) often led to stakeholders becoming unmotivated to be involved, with it being said that “*you're getting paid per hour to go out and deliver a program, and then they want you to do more admin on top of that” (SC 5).* Other identified challenges related to intervention delivery were volunteer dependency, lack of access to “trained” or specific personnel, and high levels of personnel turnover (i.e., losing expertise or experience).

*“One of the challenges with any volunteering structure, with volunteer turnover, if one good champion steps aside due to burn out, illness, work obligations or family commitments, or just needs a break, the whole deck of cards can collapse if it isn't built into the structures of the club.” (SC 3)*

### Implementation resources and supports

Provision of education and training opportunities were seen as important, as was the need for this to be an iterative process; *“You don't just train someone and let them off and then get annoyed when they don't do it exactly as you told them to do it. You let them off, you bring them back, you learn, you check in” (RE 6)*. Additionally, participants noted that implementation manuals, personnel support, and an intervention website were resources that aided intervention delivery.

“Those resources attract an awful lot more schools into the process, because we're asking them to do something extra…but we're giving them things to make it easier to do so.” (SC 12)

However, when these resources and supports are not available, unclear, or seen as not fit for purpose, challenges occurred.

*“We realized after that; the teachers didn't really use it [PA directory resource]. They really didn't. So, while these guys said, ‘Yeah, it's a good idea’…they actually didn't use it. So, we realized for scaling it up that that was a complete waste of time.” (SC7)*

### Advertisement and branding

Building a recognisable brand and using consistent language were viewed as implementation facilitators, especially useful for recruitment of new locations and participants. The size of Ireland and established networks were mentioned as aspects that facilitate advertisement of interventions, but what is advertised needs to be positive. Lack of consistent messaging or a “challenge” being associated with delivery of the intervention (e.g., high administration workload) were mentioned as factors that led to implementation barriers.

“For a campaign to be successful on a national level, you need to make sure that the communication is clear, with people who have the upper ground [decision makers]” (PM 5).

#### Reflection and Evaluation

Participants described a need to ensure reflection and evaluation was embedded within the different stages of implementation, facilitating future buy-in, support and decision making. A “lack of evidence base”, either academic or non-academic, was seen as a barrier for implementation by many. This was perceived as important during the planning of the intervention but also at later stages when re-applying for funding or planning for intervention scale-up.

“In health promotion, we would have lots of good ideas about projects, but unless we have the evidence to support them, or insert in a project that there’s a commitment to funding for the evidence, then it’s really impossible to scale them up.” (PM 1).

During intervention delivery, ongoing evaluation and use of feedback loops were seen as necessary for ensuring adequate updating of the intervention.

*“Over the years, the* <*intervention name*> *has responded to what schools need and has listened to teachers and listens to the system…So really is at the cutting edge I think of being relevant and of being of value.” (PM 3)*

It was noted that failure to update an intervention led to service providers and users becoming “bored” but on the other hand, a fundamental change could be detrimental to future implementation, such as adding a charge to a previously free intervention.

#### Intervention scale-up

Participants viewed intervention scale-up as a challenging process, where balancing the adaption of an intervention with ensuring fidelity needs to be considered. Planning for scale-up from the outset was seen as essential as it helped identify what resources and supports would be needed. Aspects mentioned were reflective of “practical considerations” covered during the planning stage, including assessment of the context and setting for new locations, identifying partnerships, and ensuring adequate personnel and resources were available for increased capacity related to delivery. Several participants noted the use of a phased approach in combination with a clear framework for implementation helped improve fidelity and ensure adequate resources were available during scale-up. Coordinator 3 stated that they *“received 208 applications for phase four and we accepted in 120 clubs and that was just down to capacity…That phased approach to it was our solution. You know that each phase we'd bring on X number of clubs, because we just didn't have capacity or the manpower to work closely and ensure the efficacy of the model.”*

#### Commitment, engagement and support

Participants felt that voluntary or formal commitment and engagement from different stakeholders, funding, and recognition of good practice was needed throughout the different stages of implementation.

### Lead organisation/ stakeholder support

The importance of identifying relevant stakeholders and agencies, both within and external to the intervention team, and identifying their role throughout the implementation was highlighted. It was felt that ensuring these were identified provided knowledge and expertise that overcame several practical challenges.

“Those broad partnerships that were developed along the way was the key. Stakeholders, and I don't mean on the ground stakeholders, I mean the agencies around us.” (SC7)

However, challenges with gaining and maintaining buy-in from other agencies such as competing priorities, lack or change of “key driving person”, and lack of formalised agreements between agencies were also acknowledged. Participants noted the importance of a communication plan or strategy to help raise awareness and engage relevant stakeholders. This could include the establishment of networks, and use of key events, media and publications to ensure effective multi-sectoral and multi-level communication. Effective communication was observed as essential for the sustainability of any intervention, enabling the best use of resources through the sharing of knowledge and best practice.

*“We have a communication channel for all the ambassadors in the country, and if someone saw a really good idea, we might hear of some really good idea that someone has…which otherwise you may not hear of.” (SP 6)*

### Community support

Generating community engagement was observed as essential for most interventions with challenges noted where communities were resistant to change. Several participants emphasized the need to raise awareness and build capacity at a community level to help generate buy-in and allow community ownership of the intervention, leading to sustainability. PM6 felt “y*ou can develop the most fantastic initiative with lots of evidence generated from around the country about how successful it would be but if the community doesn't buy into it or believe in it, it won't happen”.* Strategies to involve the community included communicating with pre-established community groups and involvement of the community when developing and implementing the intervention.

### Research partnership

Participants highlighted that a research partnership overcame challenges related to evaluation such as the lack of knowledge regarding data collection and analysis for demonstrating impact and future decision making.

“We’ve commissioned research for both primary and post-primary [schools]…And they have been two of the best things that have ever happened. I suppose, you move forward only on the basis of your knowledge as to how already it's impacting on the ground.” (PM3).

Challenges associated with research partnerships included the time required for completion of a “research project”, blinkered or narrow view of researchers, and translating academic research into practice.

“We're very keen to hear research but what we see as a really big challenge is translating that research into practical implementation. And the flexibility.” (PM 6)

### Funding support and timeframes

Funding support for the initiation, maintenance and scale-up of an intervention was identified as all requiring consideration. At a funder level, a clear model that shows decision processes, vision, purpose of fund, expectancies of awardee (including monitoring of impact) and amount of funding available all facilitate the process. Also understanding the timeframe required to report short-, intermediate- or long-term outcomes is helpful.

“We are clear about the amount of funding that we provide to them [potential applicants], and we’re clear on the level of service delivery that is expected for that, and [intervention name] report on that monthly.” (FU 4).

At the intervention level, identifying funds that align with the intervention purpose, demonstrating an evidence base and need, and in some cases, progress from past funding all help to secure funding.

“[Intervention name] had such an amount of research done on it, that it made perfect sense and it was kind of in line with our values and ethos that made sense” (FU 1)

Lack of available funding, long-term funding commitments, and a clear funding model were all reported as challenges. Conditions that come with funding were often seen as challenging to the awardee and include a short time frame to meet funding objectives and pressure to demonstrate impact.

“The pressure to get funding spent often leads to work being completed, but not in the planned way that it needs to happen.” (SC 9)

Participants noted that a lack of funding support to ensure effective advertisement and impact evaluation resulted in challenges for other areas noted as important for successful implementation.

“They [funders] believe that the fluffy stuff like communications is just all kind of extra. When in actual fact, if you don’t do the communication alongside the initiative, it won’t work”. (PM 6)

### Recognition

Participants emphasised the role of recognition facilitating intervention implementation. Recognition experienced by participants can be distilled into recognition for 1) the intervention and its achievements and 2) those involved in delivery and support. Recognition for the intervention was shown through awards (national and international), media publications (e.g., good news stories), and showcasing work done through the lead organisation at events, meetings and on social media. Recognition for those involved in delivery and support was through annual events, media publications, engagement with personnel from lead organisation, and from the service users.

*“It's incredible them [who the service user?] getting the flag and plaque, what it means to them and that they can go back to their club executive, the project team and say, "Here you are, this is what our work has achieved." (SC 3)*

Participants believed that recognition of the intervention achievements helped generate future buy-in and support, especially financial, while acknowledging the work of those delivering and supporting at a local level improved personnel retention. SC1 noted “*we won that award for* < *intervention name* > *…we did our own bit of PR around it, but I suppose from a national perspective and from even a potential funding perspective it's always great to have those things on your side.”*

## Discussion

The purpose of this study was to examine different stakeholder’s perceived factors related to the implementation of PA interventions in Ireland. Findings revealed that stakeholders perceive implementation to occur over four stages: planning, delivery, reflecting and evaluating, and scale-up. Similar to the CFIR (see Fig. 1), the stages relate to the processes of planning, executing, and reflecting and evaluating. The process of engaging and involving relevant individuals [21] was seen as occurring across all stages as displayed in “commitment, support and engagement”. Participants also noted an additional stage of implementation that related to the scale-up of an intervention, which was perceived to experience its own barriers and facilitators as discussed later.

The stages identified include separate processes and activities that are inter-related with one another. For example, communication between stakeholders within the inner and outer setting can impact understanding of context and available resources.

### Planning the intervention.

When planning the intervention, generating evidence (i.e., research driven) and a need (i.e., practice/policy driven) for the intervention was important for gaining initial support and buy-in from the relevant stakeholders. Our previous systematic review identified that a lack of any evidence base acts as a barrier for overall implementation of community-based PA intervenitons [23]. The current study also identified the need for an intervention to be usable in practice, whereby it needs to be adaptable for changes in context. This need for intervention adaptability based on context or setting has also been identified in other studies [23, 24, 27]. However, while acknowledging the importance of adaptability, our previous review found that adaptability could also act as an implementation barrier due to it having a negative influence on fidelity [23]. A suggested strategy, which is noted in the literature [35], is the collaborative development of a logic model to clearly present the overarching outcome of an intervention and detail the active inputs and processes needed to achieve it. Of importance is being able to achieve the intervention outcome in different ways can provide flexibility, aiding delivery in different contexts. It is also important to consider access to personnel with the relevant experience and expertise, accessibility to resources (e.g., facilities and equipment), and mechanisms to deal with administration burden, which have been noted as factors related to implementation success in the literature [23].

#### Delivery of the intervention

At the delivery stage, the importance of an organisational structure and adequate personnel to support the various activities (e.g., delivery, advertising, administration) was evident. Within these organisational structures, the need for a steering committee and “champions” at multiple levels to advocate for the intervention were noted. Koorts and colleagues (2018) highlight that these individual and organisational champions aid intervention implementation and sustainability and advise the use of participatory approaches when planning to identify these potential champions [25]. Within the organisational structure, having a multi-disciplinary steering committee with expertise across the stages of implementation, increases the success for intervention delivery. Essential to this is effective communication across sectors and between levels of the organisational structure. Failure to establish effective communication during the planning and delivery stage can lead to the growth of challenges that could otherwise be avoided. This finding is in keeping with previous research that highlights the importance of communication for implementation success, showing that when it is effective it can be a facilitator but when it is lacking it can be a barrier [23, 24, 27]. Ensuring effective communication between stakeholders, within and across interventions, can also avoid duplication of activities and improve effective use of resources [36].

#### Reflection and evaluation of the intervention

Reflection and evaluation by those involved was perceived as essential and needs to be embedded within the different implementation stages, which is something that aligns with established health promotion evaluation frameworks [37]. Bauman and Nutbeam (2013) present how formative, process, impact and outcome (both intermediate and long-term) evaluation can be useful throughout the different stages of intervention implementation. Again, a logic model can prove beneficial here helping plan intervention monitoring and evaluation throughout different stages of the implementation [35]. This is seen as important for updating and adapting interventions to keep them context relevant and enjoyable for participants. Previous literature has noted the importance of intervention adaption to fit different population groups and contexts [18, 23, 24, 27]. Establishment of effective communication throughout the organisational structure can help ensure that essential updates to the intervention are translated into practice, avoiding any “missed opportunities”. However, stakeholders must consider how any intervention adaption can achieve a good fit between intervention impact and context [38]. Moore and colleagues (2021) developed the ADAPT guidance, which proposes systematic processes to aid stakeholders with adapting interventions to new contexts, and transparent reporting to facilitate understanding on what does or does not work.

#### Scale-up of the intervention

During intervention scale-up, our study found that challenges relate to ensuring effectiveness due to necessary tailoring to translate the intervention into wider contexts, which aligns with previous literature [39]. Additionally, literature highlights the importance of considering scalability of an intervention from the offset [28] and continued monitoring of intervention effectiveness during the scale-up process [18]. Participants in our study spoke about use of a step-like process to ensure that adequate resources and supports (i.e., personnel, financial, physical) were available to aid implementation while scaling up. Previous research has developed a decision support tool, the Intervention Scalability Assessment Tool, to help decision makers assess the scalability of interventions and provide guidance for researchers to design future studies [40]. Scale-up also means revising the practical considerations of delivering the intervention in wider contexts. This could be seen as a revisiting of the planning stage to ensure effective delivery on a greater scale, aligning with the CFIR, whereby each stage can be “revisited, expanded, refined, and re-evaluated throughout the course of implementation”^21, pg10^. For interventions developed within the research sector, partnership with an organisation that can manage the resources and supports required for increased capacity of scaling up was seen as helpful. However, there is still a need to advocate for PA as a valuable means of NCD prevention across the “system” to maximise such partnerships. Resources are available to help support advocacy of health priorities in national policy and funding direction. Within PA, the WHO ACTIVE technical package [41] including the promotion of PA through schools and healthcare toolkits [42, 43], and the ISPAH 8 Investments Community Hub [44]. More broadly, the Health in All Policies Framework [45] provides guidance on embedding aspects of health in decision making and implementation at national and subnational levels. The framework provides six steps to enable a health in all policies approach, which are based on aspects such as transparency, sustainability and collaboration across sectors.

#### Commitment, support and engagement

As reported in the results section, the theme of commitment, support, and engagement from relevant stakeholders was seen as overarching to the different stages of implementation. Our analysis revealed that different stakeholders, including those within the inner and outer setting, provided different types of support that could facilitate or hinder implementation. As Koorts and colleagues (2018) note, “stakeholder engagement and communications should feature throughout the implementation process, to ensure transparency in roles and responsibilities, and agreement on outcome expectations”^25, pg.6^. This highlights the importance of considering what stakeholders are required, for what role and during which stages of implementation, and clearly outlining their role in the intervention. Previous research has found that clarifying work objectives and expectations improved role clarity within government agency offices, which enhanced work satisfaction and reduced staff turnover rates [46]. However, the lead organisation or steering group need to consider the plethora of factors that influence future commitment, engagement and support from each type of stakeholder during the planning stage. As noted previously, effective communication between those in the inner (e.g., steering group) and outer settings (e.g., community partners) is essential for enabling the best use of resources and development of local knowledge, aiding implementation in different contexts and the sustainability and/or scalability of the intervention [23, 24, 27].

Another interesting aspect is the role researchers play throughout the implementation stages. Participants felt that research partnerships aided evaluation and other research focused activities, but their involvement could also lead to perceived challenges relating to the time required to conduct research and researchers having a “blinkered view”. Suggestions were made for increased collaboration between research and practice earlier in the implementation process and not only for evaluation purposes. Participants felt this would help researchers understand the intervention and acknowledge the complexity of implementing interventions in the “real world”; *“It [the intervention] doesn't go out into an ideal world, it goes out into a pragmatic world”* (RE 6). Our previous work has also shown how coordination of a network, such as I-PARC, can facilitate knowledge translation between stakeholders to understand PA promotion in Ireland [29].

#### Funding support and timeframes

The present study found that commitment, support, and engagement through the form of sustained funding was important for implementation success. Providing the option for sustained funding has also been highlighted in Australia, where Lee and colleagues (2020) found it can facilitate sustainability and build on resources already invested to scale-up interventions [28]. At a funding level, it is essential to provide clarity regarding the purpose and vision of the fund, expectations of awardee and amount of funding available, which increases transparency. Ensuring future funding and practice priorities are informed by evidence is also important. The UK What Works Network [47] demonstrates how this can be done through the collaboration of several organisations to collate and produce evidence, and disseminate this transparently to support practitioners, funders and policy makers with making evidence-based decisions around future spending. At the applicant level, it is important to identify funds that align with the intervention purpose and demonstrate its need. Furthermore, when seeking sustainable funding it is vital to show continued progress and impact of the intervention.

#### Recognition

Commitment, support and engagement was also felt through recognition of intervention achievements and for those involved in its delivery. This finding is similar to previous workplace research showing that when employees receive recognition from managers, they are more likely to invest their efforts around that work [48]. Other research showed that volunteers within a service organisation felt that recognition, ranging from unplanned conversations with a manager to planned events, were a benefit of their work and they valued it [49]. It is recommended that lead organisations provide opportunities to show recognition to help highlight 1) intervention achievements, which help gain future support, and 2) personal achievements, which help with personnel retention. However further research is needed to understand how different types of recognition influence future support for PA interventions.

#### Strengths and limitations

A key strength of this study is the inclusion of practitioners, funders, researchers and policy makers involved with the implementation of PA interventions within different domains. The use of data triangulation, using different stakeholders’ perceptions increases the validity of the study findings [50, 51] and helps generate a richer picture regarding the factors related to implementation and scale-up. Despite all participants of this study originating from the island of Ireland, findings can be useful for international readers. To help readers apply this work to other countries and contexts, information regarding the current PA context in Ireland, participants and interventions included, and how these findings relate to previous research [50] are provided. There are several limitations to this study that need to be acknowledged. First, the study did not include intervention participants who could have provided another perspective regarding the factors related to implementation and is something for future research to consider. Secondly, the use of a qualitative design means that the data is representative of participants’ perceptions of the intervention they helped support, coordinate, or deliver. Multiple coders were used in the analysis to avoid assumptions being made by the lead author and increase rigour in the methods, while reflexivity was used by the lead author to recognise how their values and views may influence the findings adding further credibility to this research and a true representation of the data [51, 52]. Finally, only three (27%) of the interventions included were implemented in Northern Ireland (in addition to the Republic of Ireland). Future research needs to consider the value of using an all-island approach, with more representation from work in Northern Ireland, when understanding the implementation and scale-up of PA interventions.

## Conclusion

The findings of this study add to the previous knowledge base, exploring perceived factors related to the implementation and scale-up of PA interventions in Ireland. These findings reveal the importance of the planning stage for developing an evidence base, establishing an organisational structure and effective lines of communication, and identifying strategies to overcome potential challenges. Furthermore, it is important to identify what stakeholder groups are required to support the implementation, when they are required and in what role, allowing each stakeholder group to understand what is expected of them as part of the implementation team. Gaining funding support was seen as a challenge but could be aided through a clear funding structure from the funder, and demonstration of the evidence and how it aligns with the aims of the fund by the applicant. In conclusion, this research highlights multiple factors, that are inter-related, that influence the implementation of PA interventions, but also identifies many strategies that can be utilised to enable greater likelihood of success (Table 3).

**Table 3:** Suggested recommendations based on the study findings

Stages of Implementation	Suggested recommendations
Intervention Planning	• Demonstrate need (national and/or local need) • Use logic models to identify intervention outcomes and plan ways they can be achieved (e.g. aiding flexibility for different contexts) • Ensure any planned cost to participants is justified • Involve all relevant stakeholders in the planning stage
Intervention Delivery	• Identify “champions” to support implementation at different levels of the organisational structure • Ensure relevant implementation resources (e.g. manuals, personnel, website) meet the needs of the target audience • Develop effective communication methods (e.g. meetings, reports) to facilitate feedback for decision making
Reflection and Evaluation	• Embed reflection and evaluation into different stages of implementation • Use logic models to plan different forms of evaluation (i.e. formative, process, impact, outcome) throughout implementation stages • Use effective communication to feedback evaluation findings and ensure relevant updating/adaption of the intervention
Intervention Scale-up	• Consider scalability of the intervention from the offset • Incorporate layers (i.e. national, sub-national and local) within the organisational structure to support scale-up • Use of step-like process during scale-up to ensure adequate personnel and resources are available for increased capacity needs • Identity potential partnerships with organisations that have the resources and capacity to support scale-up. This might mean advocating for the intervention impact and benefits of physical activity
**Additional Themes**	**Suggested recommendations**
Commitment, support and engagement	• Clearly present role expectations for all relevant stakeholders. This means considering what stakeholders are required, for what purpose and during which stages of implementation • Communicate the intervention plan with existing community groups to aid community buy-in • Consider research partnerships to aid with certain implementation stages but also consider ways to alleviate challenges (e.g. research language, slow process)
Funding support and timeframes	**Funder** • Develop and offer mechanisms that allow pathways for sustainable funding • Provide a clear funding plan that presents funding goal, funding available, timeframes, and applicant expectations (delivery and reporting) **Applicant** • Identify funds that align with the intervention goals • Demonstrate impact or progress to aid future funding
Recognition	Embed mechanisms, which recognise and celebrate: • Intervention achievements • Personal achievements

## Supplementary Information

**Additional file 1:** **Supplementary file 1.** I-PARCImplementation Survey 

**Additional file 2:** **Supplementary file 2.** Interview Guide – Service Provider/Coordinator

**Additional file 3:** **Supplementary file 3.** InterviewGuide – Researcher/Policy Maker/ Funder 

**Additional file 4:** **Supplementary file 4.** DeviantCodes and where they were placed within the code book 

**Additional file 5:** **Supplementary file 5.** Barriers and facilitators related to implementation and scale up of physical activityinterventions in Ireland

## Data Availability

The datasets generated during and/or analysed during the current study are available from the corresponding author on reasonable request.

## Abbreviations

PA: Physical activity
WHO: World health organization
CFIR: Consolidated framework for implementation research
I-PARC: Irish physical activity research collaboration
PM: Policy maker
FU: Funder
SP: Service provider
SC: Service coordinator
RE: Researcher
