# Supplementary material for: Factors related to the implementation and scale-up of physical activity interventions in Ireland: a qualitative study with policy makers, funders, researchers and practitioners
Source: Int J Behav Nutr Phys Act. 2023 Feb 14;20:16. doi: 10.1186/s12966-023-01413-5 (PMC9926412; doi:10.1186/s12966-023-01413-5)
Supplement: Supplementary file 1 — Additional file 1: Supplementary file 1. I-PARCImplementation Survey [file 12966_2023_1413_MOESM1_ESM.docx]

**I-PARC Implementation Survey**

| **Welcome and Introduction** |
| --- |

Welcome to the implementation survey as part of the Irish Physical Activity Research Collaboration (I-PARC). Survey 2 will take approximately 20 minutes, and it is designed to assess the barriers to and facilitators of successful PA implementation and the processes involved. Guided by the Consolidated Framework for Implementation Research (CFIR) and National Clinical Effectiveness Committee (NCEC), questions will also address issues to do with:

Initiative characteristics (source of initiative, evidence strength and quality, complexity, and cost)

Outer setting (Participant needs and resources, external networks and relationships, external policies and incentives)

Inner Setting (Structural characteristics, networks and communications, culture, and readiness for implementation)

Characteristics of the individuals involved (Motivation, self-efficacy, and individual perceptions)

Process of Implementation (Planning, engaging, executing, and reflecting and evaluating)

**Who can complete the survey?**

Individuals who understand the implementation of the initiative should complete the survey. This can include knowledge-users (i.e. service coordinators) and researchers who are involved in the initiative.

| **Consent** |
| --- |

Please check the box if you have read and understand the study information and wish to give your consent to take part in this survey.

**Q1. What initiative do you currently associate with?**

- Staying Fit for the Future
- Active School Flag
- Y-PATH
- Men on the Move
- parkrun Ireland
- Women on Water
- Enjoy Tennis
- GAA Healthy Clubs
- Active Travel Towns
- 20X20

**Q2. Where is your initiative located?**

|  |
| --- |

**Q3. Please select the personnel involved in completing this survey:**

- Sports Development Officer
- Club/Group Coach
- External Contractor
- Volunteer
- Health and Fitness Instructor
- Clinician
- Primary Care Personnel
- Other: ___________________________

| **Section 1 – Initiative Characteristics** |
| --- |

The following section looks at the characteristics of the initiative including factors such as: source of the initiative, evidence strength and quality, complexity, and cost.

**Q4. Was there supporting evidence showing that the intervention could work before implementation?**

| Yes | No | Unsure |
| --- | --- | --- |
|  |  |  |

**If so, what type, and to what extent was this evidence considered?**

|  | Not at all | A little | Some | A lot | Completely |
| --- | --- | --- | --- | --- | --- |
| Existing pragmatic research |  |  |  |  |  |
| Similar successful initiatives |  |  |  |  |  |
| Community demand |  |  |  |  |  |
| Clinical evidence |  |  |  |  |  |
| Government policy |  |  |  |  |  |
| Local policy |  |  |  |  |  |

**Q5. What are the core components of your initiative that are indispensable to its implementation? Please rank in order of importance**

| 1 |
| --- |
| 2 |
| 3 |
| 4 |
| 5 |

**Q6. Were any existing practices replaced by this initiative?**

| Yes | No | Unsure |
| --- | --- | --- |
|  |  |  |

**If so, can you expand on what exact practices were replaced by this new initiative?**

|  |
| --- |

**Q7. How difficult was it to implement this initiative? (1 = not at all to 7 = very difficult)**

| 1 | 2 | 3 | 4 | 5 | 6 | 7 |
| --- | --- | --- | --- | --- | --- | --- |
|  |  |  |  |  |  |  |

**Q8. What level of costs were incurred in implementing this intervention?**

| Low | Medium | High |
| --- | --- | --- |
|  |  |  |

**Were these costs higher, on target, or lower than expected?**

|  |
| --- |

**Q9. To what extent would you rate cost as a barrier or facilitator to effective implementation of your initiative? (1 = barrier to 7 = facilitator)**

| 1 | 2 | 3 | 4 | 5 | 6 | 7 |
| --- | --- | --- | --- | --- | --- | --- |
|  |  |  |  |  |  |  |

**Please expand on your answer**

|  |
| --- |

| **Section 2 – Outer setting** |
| --- |

The following section looks at the outer setting of the initiative including factors such as: Participant needs and resources, external networks and relationships, and external policies and incentives.

**Q10. When planning the opportunity, did you work with any of the following people or groups? (Please rate each option)**

|  | Not at all | Consulted | Part of team |
| --- | --- | --- | --- |
| Organisations or agents delivering the intervention |  |  |  |
| Target group or its representative |  |  |  |
| Other community groups/non-government organisations |  |  |  |
| Clinical groups/Practitioners |  |  |  |
| Government agencies |  |  |  |
| Private Sector/Industry |  |  |  |

Other: _______________________________________________________

**Q11. To what extent does this initiative meet the needs and preferences of participants? (1 = not at all to 7 = completely)**

| 1 | 2 | 3 | 4 | 5 | 6 | 7 |
| --- | --- | --- | --- | --- | --- | --- |
|  |  |  |  |  |  |  |

**Q12. What is the level of information exchange/networking that staff have with others outside of this initiative?**

| Low | Medium | High |
| --- | --- | --- |
|  |  |  |

**Q13. To what extent are external partnerships/relationships important to the success of this initiative? (1 = not at all to 7 = extremely important)**

| 1 | 2 | 3 | 4 | 5 | 6 | 7 |
| --- | --- | --- | --- | --- | --- | --- |
|  |  |  |  |  |  |  |

**Q14. What kind of information exchange/networking do staff have with others outside their setting?**

|  |
| --- |

**Q15. To what extent are external policies, regulations or guidelines supporting/facilitating the implementation of this initiative? (1 = not at all, 7 = extremely supportive)**

| 1 | 2 | 3 | 4 | 5 | 6 | 7 |
| --- | --- | --- | --- | --- | --- | --- |
|  |  |  |  |  |  |  |

**Please list any specific ones:**

| 1 |
| --- |
| 2 |
| 3 |
| 4 |
| 5 |

| **Section 3 – Inner setting** |
| --- |

The following section looks at the inner setting of the initiative including factors such as: Structural characteristics, networks and communications, culture, implementation climate, readiness for implementation.

**Q16. How many years has the organisation operating the initiative been in existence?**

| 0-5 years | 6-10 years | 11-15 years | 16-20 years | 21-25 years | 25 years + |
| --- | --- | --- | --- | --- | --- |
|  |  |  |  |  |  |

**Q17. Were any changes to the organisational structure needed to accommodate the initiative?**

|  |
| --- |

**Q18. What is the level of staff turnover at your organisation?**

| Low | Medium | High |
| --- | --- | --- |
|  |  |  |

**Q19. To what extent does geographic spread of the initiative act as a barrier or facilitator to successful implementation? (1 = barrier to 7 = facilitator)**

| 1 | 2 | 3 | 4 | 5 | 6 | 7 |
| --- | --- | --- | --- | --- | --- | --- |
|  |  |  |  |  |  |  |

**Q20. To what extent does the quality of communication act as a barrier to or facilitator of this initiative? (1 = barrier to 7 = facilitator)**

| 1 | 2 | 3 | 4 | 5 | 6 | 7 |
| --- | --- | --- | --- | --- | --- | --- |
|  |  |  |  |  |  |  |

**Q21. Did all staff have access to training and information about how to implement this initiative?**

| None | A few | Some | Most | All |
| --- | --- | --- | --- | --- |
|  |  |  |  |  |

**Q22. How do staff find out about best practice and accomplishments?**

|  |
| --- |

**Q23. To what extent are new ideas embraced and used to make improvements? (1 = not at all to 7 = completely)**

| 1 | 2 | 3 | 4 | 5 | 6 | 7 |
| --- | --- | --- | --- | --- | --- | --- |
|  |  |  |  |  |  |  |

**Q24. How well does the initiative fit with the existing work culture and practices?**

| Not at all | Mostly not | Slightly not | Neutral | Somewhat | Mostly | Completely |
| --- | --- | --- | --- | --- | --- | --- |
|  |  |  |  |  |  |  |

**Q25. Did you have the necessary resources and support to implement this initiative?**

| Yes | No | Unsure |
| --- | --- | --- |
|  |  |  |

**If not, can you expand on what other resources and support you needed?**

|  |
| --- |

| **Section 4 – Characteristics of Individuals** |
| --- |

The following section looks at the characteristics of individuals in the initiative including factors such as: Individual capacity – physical and psychological and motivation.

**Q26. To what extent are you motivated to implement the initiative? (1 = not at all to 7 = extremely)**

| 1 | 2 | 3 | 4 | 5 | 6 | 7 |
| --- | --- | --- | --- | --- | --- | --- |
|  |  |  |  |  |  |  |

**Q27. To what extent do you feel confident in your ability to implement the initiative? (1 = not at all to 7 = extremely)**

| 1 | 2 | 3 | 4 | 5 | 6 | 7 |
| --- | --- | --- | --- | --- | --- | --- |
|  |  |  |  |  |  |  |

**Q28. Do you identify with the goals of the initiative? (1 = not at all to 7 = completely)**

| 1 | 2 | 3 | 4 | 5 | 6 | 7 |
| --- | --- | --- | --- | --- | --- | --- |
|  |  |  |  |  |  |  |

**Q29. Are you satisfied with your role and responsibilities?**

| Not at all | Mostly not | Slightly not | Neutral | Somewhat | Mostly | Completely |
| --- | --- | --- | --- | --- | --- | --- |
|  |  |  |  |  |  |  |

**Q30. Do you feel that the organisation could be doing things more efficiently?**

| Yes | No | Unsure |
| --- | --- | --- |
|  |  |  |

**If so, can you expand on how they could be doing things more efficiently?**

|  |
| --- |

| **Section 5 – Process of Implementation** |
| --- |

The following section looks at the process of implementation of the initiative including factors such as: planning, engaging, executing, and reflecting and evaluating.

**Q31. How long was the planning stage before implementation of this initiative?**

| 0-6 months | 6-12 months | 12-18 months | 18-24 months | 24 months + |
| --- | --- | --- | --- | --- |
|  |  |  |  |  |

**Q32. Was the planning of this initiative guided by any tool or framework?**

| Yes | No | Unsure |
| --- | --- | --- |
|  |  |  |

**If so, can you list them?**

|  |
| --- |

**Q33. Did you pilot this initiative before launching it?**

| Yes | No | Unsure |
| --- | --- | --- |
|  |  |  |

**If so, can you expand on how you piloted it?**

|  |
| --- |

**Q34. Did your organisation recruit any champions to support/promote your initiative?**

| Yes | No | Unsure |
| --- | --- | --- |
|  |  |  |

**If so, was this a priority?**

| Not at all | Low priority | Medium priority | High priority |
| --- | --- | --- | --- |
|  |  |  |  |

**Q35. To what extent is evaluation embedded in the practices of this organisation? (1 = not at all to 7 = completely)**

| 1 | 2 | 3 | 4 | 5 | 6 | 7 |
| --- | --- | --- | --- | --- | --- | --- |
|  |  |  |  |  |  |  |

**Q36. How often does reflection and evaluation take place?**

| Never | Sometimes | Often | All the time |
| --- | --- | --- | --- |
|  |  |  |  |

**Q37. How is feedback communicated with staff? (Please tick all applicable)**

|  |
| --- |

| **Thank you & Finish** |
| --- |
