# Supplementary material for: Factors related to the implementation and scale-up of physical activity interventions in Ireland: a qualitative study with policy makers, funders, researchers and practitioners
Source: Int J Behav Nutr Phys Act. 2023 Feb 14;20:16. doi: 10.1186/s12966-023-01413-5 (PMC9926412; doi:10.1186/s12966-023-01413-5)
Supplement: Supplementary file 2 — Additional file 2: Supplementary file 2. Interview Guide – Service Provider/Coordinator [file 12966_2023_1413_MOESM2_ESM.docx]

**Interview Guide – Service Provider/Coordinator**

Date for interview:_________________________________________________

Location:_________________________________________________

Respondent(s):___________________________________________

Interviewer:_____________________________________________­­­­­_________

Reporter: ­­­­­­­­­­­­­­­­­­­­­­­­________________________________________________________

**Introduction**

My name is … and I am from.... I will be conducting this interview with you. The study investigates the implementation process of different interventions to learn more about the barriers and facilitators to implementing physical activity interventions. What is meant by implementation is the methods and strategies to promote the uptake of [name of intervention] into routine practice, with the aim of increasing physical activity for everyone. As part of our study, we identified 10 exemplar interventions taking place across Ireland and the knowledge-users who are key to their success.

You have been selected for the interview because of your experience and involvement in [name of intervention]. I would therefore like to ask some questions. We are interested in learning from your experiences, from now as a successful intervention, but also from what it was like at a time when it wasn’t working so well.

All the information you give to us today will remain strictly confidential. With your permission we would like to record the interview so we have an accurate record of what you have said. The interview will be transcribed and any identifying information either about you or people who you mention will be disguised to preserve anonymity.

Obviously, there are no right or wrong answers to the questions - just your perceptions and recollections of events.

If you would like to stop the interview at any time for whatever reason, just let us know. You can also withdraw your information in part or in full from the study if you change your mind about participating.

Please ensure that you have read the Participant Information Sheet before participating in the interview. The interview should take about 60 minutes - do you have any time constraints that we should know about before starting?

>> sign consent form<<

Is there anything you would like to ask me before we get started?

>> start recorder<< State your name, their name and the date

**Background and involvement in Intervention**

- Can you describe your background and how you ended up here?
- Prompts:
  - What is/was your role in the implementation process of this intervention?
  - Did you have previous experience with this type of target population program?
  - What experience do you have of the implementation of interventions?
  - What motivates you in your day-to-day role?

**Intervention development**

- Can you describe briefly the nature of the problem that the intervention was originally designed to address? (Prompt: clarity on target group + magnitude of problem)
- How was the intervention developed? Any co-creation? (Prompt: organisational change)
- How would you describe this intervention to someone outside of your organisation? (prompts: Description of the Intervention in terms of Intervention objectives, Intervention design, Underlying principles/theory, Delivery format, Components, Duration, Costs)
- Does the intervention allow tailoring to a specific context? (prompt: Core and adaptable elements, flexibility, adaptation)
  - Follow-up: Can you give me an example?
- Did the intervention change over time? If so, how and why? (prompt: adaptability)

**Implementation strategies** (prompt: different stages of implementation)

- What type of support was provided for you? (Prompt: training for staff before delivery)
- How was that received?
- To the best of your knowledge, was there a plan beforehand to put in place the intervention? (Prompt: What was your role in this?)
- Can you describe any support/ guidance for implementing the intervention according to how it was planned? (Prompt: fidelity; support during intervention implementation)
- Any other implementation strategies/ materials?
- Did that change over time? If so, how and why?

**Important actors** (prompt: different stages of implementation)

- Who were important people in the implementation process? (Prompt: community, local government/council)
- Why were they important?
- What, if anything, hindered the process?
- Did that change over time? If so, how and why?

**Impact and outcomes** (prompt: different stages of implementation)

- Overall, who do you think the intervention reaches? (Prompt: was it as expected or lower/higher)
- In your opinion, what was the impact on the physical activity levels of the participants?
- What did the people who used this intervention think of the intervention (process evaluation)?
  - How is this feedback used?

**Facilitators for implementation** (prompt: different stages of implementation)

- Was there anything that helped or enabled you to implement the intervention? On the intervention level, individual level, provider level, organizational level, community level? (Prompt: examples of each of the levels)
  - Provider – focus on provider level, individual level, community level
  - Key areas: funding and sustainability at both intervention level and personal level (eg. financial remuneration)
- Did that change over time? If so, how and why?

**Barriers for implementation** (prompt: different stages of implementation)

- Were there any barriers when implementing the intervention? On the intervention level, individual level, provider level, organizational level, community level? (Prompt: examples of each of the levels)
  - Provider – focus on provider level, individual level, community level
  - Key areas: funding and sustainability at both intervention level and personal level (eg. financial remuneration)
- How did you overcome these barriers?
- Did that change over time? If so, how and why?

**Future**

- Are there any planned modifications for this intervention?

**Closing**

- Is there anything else you think is important that we have not talked about?
- Thank interviewee

>> stop recorder<<
