# Supplementary material for: Factors related to the implementation and scale-up of physical activity interventions in Ireland: a qualitative study with policy makers, funders, researchers and practitioners
Source: Int J Behav Nutr Phys Act. 2023 Feb 14;20:16. doi: 10.1186/s12966-023-01413-5 (PMC9926412; doi:10.1186/s12966-023-01413-5)
Supplement: Supplementary file 3 — Additional file 3: Supplementary file 3. InterviewGuide – Researcher/Policy Maker/ Funder [file 12966_2023_1413_MOESM3_ESM.docx]

**Interview Guide – Researcher/Policy Maker/ Funder**

Date for interview:_________________________________________________

Location:_________________________________________________

Respondent(s):___________________________________________

Interviewer:______________________________________________________

Reporter: ________________________________________________________

**Introduction**

My name is … and I am from.... I will be conducting this interview with you. The study investigates the implementation process of different interventions to learn more about the barriers and facilitators to implementing physical activity interventions.

- What is meant by implementation is the methods and strategies to promote the uptake of [name of intervention] into routine practice, with the aim of increasing physical activity for everyone.
- Scale up is described as replicating and extending the reach of an intervention into other localities, cities, or regions.

As part of our study, we identified 10 exemplar interventions taking place across Ireland and the knowledge-users who are key to understanding them. .

You have been selected for the interview because of your experience and involvement in [name of intervention]. I would therefore like to ask some questions. We are interested in learning from your experiences of implementing and scaling up physical activity interventions, and the support structures in place.

- All the information you give to us today will remain strictly confidential. With your permission we would like to record the interview so we have an accurate record of what you have said. The interview will be transcribed and any identifying information either about you or people who you mention will be disguised to preserve anonymity.
- Obviously, there are no right or wrong answers to the questions - just your perceptions and recollections of events.
- If you would like to stop the interview at any time for whatever reason, just let us know. You can also withdraw your information in part or in full from the study if you change your mind about participating.
- Please ensure that you have read the Participant Information Sheet before participating in the interview. The interview should take about 60 minutes - do you have any time constraints that we should know about before starting?

>> sign consent form<<

Is there anything you would like to ask me before we get started?

>> start recorder<< State your name, their name and the date

**Background**

- In your own words can you please describe your current role? [Policy maker; Both policy maker and researcher; Practitioner/Service Manager; Consultant; Funder]
- Can you describe your previous experience with physical activity promotion/interventions
  - - Prompt: years experience, development, implementation, and/or scale-up of population health interventions?
    - What do you feel you have spent most of your time working with (i.e. development, implementation, and/or scale-up).

**Decision processes** (Decision processes are processes by which decision makers identify information, evaluate alternatives, and make decisions on courses of action.)

- Can you share with me how you have been involved in the decision processes to implement and/or scale up physical activity interventions into broader policy or practice? (Prompt, if required: Refer back to definition of implementation or scale-up from introduction.
- In the past five years, how many times have you been involved in decision processes to implement and/or scale up physical activity interventions?
  - Are there certain qualities or characteristics you look for when looking to 1) implement and/or 2) scale-up interventions?
  - Now, thinking specifically about **<insert intervention name>**; were you involved in a decision process to implement and/or scale up.  Could you briefly describe the intervention?
  - Was the intervention being implemented for the first time, or being scaled up?
  - What formal processes, if any, were undertaken to inform decisions to implement and/or scale up the intervention (or not)? (Prompts if required: stakeholder consultation, expert consultation, Advisory Group, meetings of Ministers, etc.)
  - What role did you personally have in the decision processes? (Prompts if required: Policy maker, advocate, expert, advisor, etc.)
  - How or by whom was the final decision to implement and/or scale up the intervention (or not) made? (Prompts if required: political process, policy process, an individual such as a Minister or senior bureaucrat, by a Government/s, research institute)
  - What factors and information sources, if any, enabled decision-making about whether to implement and/or scale up the intervention or not?
  - What (if any) influence did research evidence have on the decision processes?
    - If research evidence did influence the decision processes, what type of research was used and how was it applied? (Prompt if necessary: determinants research, formative research, measurement research, intervention research, economic evaluation, etc.)
    - How would you describe the level of support in using this evidence?
  - What factors were barriers to decision-making about whether to implement/and or scale up the intervention or not? (Prompt: support/resources, evidence-base).
  - What were the final outcomes of these decision processes? (Prompt if necessary: intervention was scaled up straight away, more info needed, intervention abandoned, etc.)
  - Was this process typical? If not, can you describe how it differed from other processes (if any) you have been involved in previously.

**Overall roles of policy makers, researchers and funders**

- From your experience, what roles do policy makers, researchers and funders play in the process of implementing and/or scaling up physical activity interventions? (Prompts in required: how do they differ and why?)
- From your experience, what are the most powerful influences on decisions to implement and/or scale up physical activity interventions?
  - In your opinion, which should be the most important influences on decisions to implement and/or scale up population health interventions?

**Personal experiences of the scale up process of physical activity interventions into broader policy and practice.**

- Have you ever been responsible for implementing a process of scaling up a physical activity intervention into broader policy or practice? (Prompt, if required: by scaling up we mean taking a health intervention shown to be efficacious on a small scale and/or under controlled conditions and expanding it under real world conditions into broader policy or practice.) [Yes/No]
  - From your experience, what do you think are the key barriers to effectively scaling up physical activity interventions into broader policy and practice? (prompt: support)
  - From your experience, what do you think are the key success factors in scaling up population health interventions into broader policy and practice? (prompt: support, partnership and what is meant).

**Future scale up**

- Are there any future intentions to (further) scale up this **<insert intervention name>**?
- Are the necessary support structures in place? What would need to happen to allow for the (further) scale up of this intervention? (Prompt: funding call/source, policy changes, research evidence base, etc)
