# Supplementary material for: Factors related to the implementation and scale-up of physical activity interventions in Ireland: a qualitative study with policy makers, funders, researchers and practitioners
Source: Int J Behav Nutr Phys Act. 2023 Feb 14;20:16. doi: 10.1186/s12966-023-01413-5 (PMC9926412; doi:10.1186/s12966-023-01413-5)
Supplement: Supplementary file 4 — Additional file 4: Supplementary file 4. DeviantCodes and where they were placed within the code book [file 12966_2023_1413_MOESM4_ESM.docx]

Supplementary File 4. Deviant Codes and where they were placed within the code book

| **CFIR Domain** | **Deviant Factor** | **Decision made after team discussion** |
| --- | --- | --- |
| Intervention Characteristics | Workload required | Merged with “Usability of Intervention” |
| Intervention Characteristics | Intervention content | Renamed “Conflicting views on intervention content” and “Clear criteria for intervention delivery” |
| Intervention Characteristics | Intervention track record | Merged with “Research/Evidence Base” |
| Intervention Characteristics | Participant perception of intervention | Removed from code book |
| Characteristics of Individuals | Respected Individuals | Removed from code book |
| Characteristics of Individuals | Providers perception of why participants were involved | Removed from code book |
| Outer Setting | Timing (i.e. luck) | Removed from code book |
| Outer Setting | Publication of work | Merged with “Recognition” (Inner Setting) |
| Outer Setting | Access to Facilities | Merged with “Practical Considerations” (Intervention Characteristics) |
| Outer Setting | Organisational concern with risk related to supporting intervention | Removed from code book |
| Inner Setting | Change in driving person | Merged with “Organisational Structure” |
| Processes of Implementation | Data Systems | Removed from code book |
| Processes of Implementation | Quality and use of data | Merged with “Data Collection” |
| Processes of Implementation | Solution Generation | Merged with “Needs Assessment” |
