# Supplementary material for: Factors related to the implementation and scale-up of physical activity interventions in Ireland: a qualitative study with policy makers, funders, researchers and practitioners
Source: Int J Behav Nutr Phys Act. 2023 Feb 14;20:16. doi: 10.1186/s12966-023-01413-5 (PMC9926412; doi:10.1186/s12966-023-01413-5)
Supplement: Supplementary file 5 — Additional file 5: Supplementary file 5. Barriers and facilitators related to implementation and scale up of physical activityinterventions in Ireland [file 12966_2023_1413_MOESM5_ESM.docx]

**Supplementary File. Barriers and facilitators related to implementation and scale up of physical activity interventions in Ireland.**

| Domain | Facilitator | Barrier |  |
| --- | --- | --- | --- |
| 1. **Intervention Stages** | | |  |
| - 1. **Intervention Planning**   This stage involves the planning of the intervention itself and other practical considerations. This planning helps overcome future challenges and ensure the intervention can be feasible in practice, sustainable and scalable. Challenges related to the planning stage relate to lack of stakeholder involvement, needs assessment or identifying solutions to a new problem. | | | |
| ***The Intervention*** | | | |
| Evidence | The intervention is based on either academic or non-academic evidence. Ensuring reflection and evaluation are embedded within the implementation stages can aid this. | Intervention has a lack of evidence regarding impact and implementation, which leads to a lack of support. |  |
| Need for Intervention | There is a demonstrated need for the intervention from national data or policy, personal experiences, or appetite of the sector. Conducting a needs assessment as part of the intervention planning can aid this. | None noted |  |
| Cost | Cost of intervention needs to be considered during the planning stage. Low financial cost to participant and coordinating agency is seen as a facilitator. If there is a cost for the stakeholder or participant, value needs to be demonstrated and any financial gain should be used to maintain or progress the intervention. | Costs to the stakeholder such as venue hire, equipment and advertisement of the intervention, and cost for the participant to attend. |  |
| Usability of Intervention | Intervention is fit for target population, adaptable for use in different contexts, compatible with current practices and is feasible to set up and implement. | Intervention is not appealing to target population, content is too prescriptive, does not align with funder or coordinator aims. or is complex to deliver in practice. |  |
| ***Practical Considerations*** | | | |
| Accessibility of facilities | Easy access to safe facilities that can be used to deliver the intervention. | Lack of access to facilities due to competition, structure of facility (no disability access), or location of facility (i.e., parking, long distance from homes). |  |
| Context/Setting | Observing aspects related to any change in location/setting, organisation support (e.g., change in policy, existing organisations, curriculum), and uncontrollable factors (e.g. weather, competing interests). | Failing to plan strategies to overcome challenges within the context of the “intervention”, such as the delivery location, policy support, administration (e.g., insurance, data protection, funding applications), competing interventions, and uncontrollable factors (e.g., weather). |  |
| Personnel | Access to adequate numbers of “trained” personnel to deliver and support the intervention implementation. | Intervention is dependent on volunteers, lacks specific personnel or personnel turnover is high. |  |
| Admin Burden | Adequate systems, capacity and support available to help make administration tasks easier. | Multiple “sign-off” levels, handling queries from participants, completing evaluation forms and completing essential paperwork (e.g., safeguarding). |  |
| - 1. **Intervention Delivery**   Strategies noted to facilitate intervention delivery include use of a recruitment strategy, meetings (i.e., information, follow-up and renewal), leaders at multiple levels, and the use of clear content and participant criteria. Challenges noted during this stage related to recruitment of tutors and participants, lack of clarity on screening/registration process, catering different levels of ability and a lack of capacity to include a social aspect. | | | |
| ***Organisational Structure and Staffing*** | | | |
| Organisational Structure | Evident structure within lead organisation, including a steering group or person and specific personnel to support various aspects of implementation (e.g., communications, core delivery, risk insurance). A layered organisational approach can aid the scale up of interventions (i.e., adding additional layers of support and resources). | None noted. |  |
| Role Responsibility | Agreement and understanding of stakeholders are essential for ensuring role clarity when implementing the intervention. | Not establishing roles when planning the intervention can lead to difficulty with establishing the buy-in of each stakeholder and sometimes lead to no “key driver”. |  |
| Collaboration and Communication | A communication plan or strategy aids the implementation of an intervention and helps to overcome challenges. This can include the establishment of networks and use of key events, media and publications in order to share knowledge and ensure communication between stakeholder organisations. | Lack of any communication plan for stakeholders and between interventions in similar populations can lead to poor decision making and knowledge sharing. |  |
| Experience and Expertise | Those involved have experience and expertise in the topic area, the target population or at skills at different stages of implementation. Different expertise can come from different people within the team. | Lacking experience due to broad focus of an individual’s job or constant change in personnel. |  |
| Attitudes and Views | Those involved value that the intervention works in practice, benefits participants or there is a proven need. | Lack of knowledge regarding the intervention, its purpose or target group can lead to a lack of value by those involved. |  |
| Motivation | Those involved are motivated by personal experiences, viewing it as a fulfilling experience, consistent income or feeling a responsibility to create change. Providing adequate supports and recognition for those involved with coordinating and delivering the intervention may foster this motivation. | Lack of support for administration requirements can lead to stakeholders becoming unmotivated to be involved with the intervention. |  |
| Time Commitment | Acknowledging the other commitments of service coordinators and providers, and highlighting what is expected regarding the location, equipment needed, and time required to deliver the intervention. | Competing interventions, jobs or hobbies create time constrains for those involved in intervention coordination and delivery. |  |
| Respected | Those developing, supporting, and delivering the intervention are respected, based on their experience and expertise by the other key stakeholder groups (e.g., funders, participants). | None noted. |  |
| ***Implementation Resources and Supports*** | | |  |
| Implementation Resources and Supports | Use of an intervention website, education and training, implementation manuals, modelling and personnel supports are common resources and supports to aid implementation. | When resources and supports are not available or they are but not seen as usable of fit for purpose challenges are created. |  |
| ***Advertisement and Branding*** | | | |
| Advertising and Branding | Building a recognisable brand with use of consistent language aid successful advertisement of your intervention. Ireland’s size (i.e., word of mouth and established networks), media, and events can be helpful advertisement tools. | A lack of advertisement consistency or a challenge associated with the intervention (i.e., high paperwork load) can have a damaging effect when recruiting key stakeholders. Other challenges related to a lack of support from funders to help develop an effective advertisement plan. |  |
| - 1. **Reflection and Evaluation** | | | |
| Reflecting and Evaluating | Planning and incorporating different forms of evaluation (formative, process, impact) that are used to build an evidence base, motivate participants and for decision making regarding the intervention’s future. | Lack of time, resources, available data systems or knowledge to evaluate make it challenging to produce evidence. |  |
| Updating Intervention | Data collected through evaluation or feedback loops is used to update and adapt the target group, content, key criteria or implementation and evaluation processes of the intervention. | Where no update occurs, participants can become “bored” but alternatively, an update to an intervention can be detrimental (e.g., adding a charge for using the intervention). |  |
| - 1. **Intervention Scale-up** | | | |
| Practical Considerations | Planning for scale-up from the beginning, identifying the need and resources/stakeholders required, using a phased approach and a clear framework or manual for implementation. | Lack of communication, evidence for the intervention, readiness of sector and support from key stakeholders when scaling-up. |  |
| Fidelity | Clarity regarding the intervention content, achievement criteria and participant inclusion criteria, and an overarching strategy to guide implementation. | Scaling-up when there is a lack of set guidelines for delivery. Variation in delivery styles by personnel can also create challenges for maintaining fidelity. |  |
| 1. **Commitment Engagement and Support** | | | |
| Lead Organisation | Commitment of organisation and those involved in implementation is evident, ensuring support and communication lines are in place as the intervention is planned, implemented and scaled-up. | Lack of internal support due to other commitments, capacity, formalised agreements, poor communication and change in “champion” at any level. |  |
| Stakeholder Support | Evidence of engagement and support at different levels aiding the implementation of the intervention. This can be from international bodies, government departments, national and funding agencies, regional, county or local bodies, those directly involved with delivering the intervention, and external bodies (e.g., GP, physios, police). Having a communication plan or strategy can aid interaction between stakeholders. | Lack of support or engagement from key stakeholders linked with an intervention. |  |
| Community Support | Buy-in from the community for the intervention can allow them to “own” it and generate sustainability. Awareness raising, communication, capacity building and collaboration all help overcome the challenges with gaining community support. | Communities where a divide exists or they are resistant to change. |  |
| Research Partnership | Involvement of researchers increases capacity for implementation and evaluation research which help increase the evidence base (e.g., publications) and provide recommendations for practice. | Involvement of research can be time consuming, lead to a constrained view of the work and create outputs that are difficult to translate into practice. |  |
| - 1. **Funding Support and Timeframes** | | | |
| Funding Support and Timeframes | At the funder level a clear funding model that shows decision processes, vision and purpose of fund, expectancies of awardee and amount available can facilitate the funding process. At the applicant level, identifying available funding, being able to demonstrate a need, evidence base, progress from previous years, and successful past working relationships help with gaining funding. | Lack of available funds, long-term funding commitments, clear funding model, and conditions that come with funding (e.g., short time frame, pressure to demonstrate impact, meeting funding objectives). |  |
| - 1. **Recognition** | | | |
| Recognition | Both the intervention and those involved are recognised through awards, celebrating individuals involved (at various levels) or through the media (e.g., breaking records, good news stories). The intervention can also be recognised by the lead organisation through showcasing (i.e., showcase work done at events, meetings, website/social media) or by important stakeholders (e.g., representative from lead agency attends launch, participants recognise work done by service provider). | Lack of recognition from 1) government agencies, 2) government departments, 3) institutions (i.e., schools, third level) for the work done to implement the intervention. |  |
